# The effect of exposure to radiofrequency electromagnetic fields on cognitive performance in human experimental studies: A protocol for a systematic review

**DOI:** 10.1016/j.envint.2021.106783

**Published:** 2021-12

**Authors:** Blanka Pophof, Jacob Burns, Heidi Danker-Hopfe, Hans Dorn, Cornelia Egblomassé-Roidl, Torsten Eggert, Kateryna Fuks, Bernd Henschenmacher, Jens Kuhne, Cornelia Sauter, Gernot Schmid

**Affiliations:** a Federal Office for Radiation Protection, Competence Centre EMF, Oberschleißheim, Germany; b Institute for Medical Information Processing, Biometry and Epidemiology (IBE), LMU Munich, Germany; c Charité – Universitätsmedizin Berlin, Corporate Member of Freie Universität Berlin and Humboldt-Universität zu Berlin, Competence Centre of Sleep Medicine, 12203 Berlin, Germany; d Federal Office for Radiation Protection, Oberschleißheim, Germany; e Seibersdorf Laboratories, Austria

**Keywords:** Specific absorption rate, Radiofrequency electromagnetic fields, High frequency electromagnetic fields, Cognitive performance, Attention, Memory, Visual task, Auditory task, Systematic review

## Abstract

**Background:**

The World Health Organization (WHO) is currently assessing the potential health effects of exposure to radiofrequency electromagnetic fields (RF-EMFs) in the general and working population. Related to one such health effect, there is a concern that RF-EMFs may affect cognitive performance in humans. The systematic review (SR) aims to identify, summarize and synthesize the evidence base related to this question. Here, we present the protocol for the planned SR.

**Objectives:**

The main objective is to present a protocol for a SR which will evaluate the associations between short-term exposure to RF-EMFs and cognitive performance in human experimental studies.

**Data sources:**

We will search the following databases: PubMed, Embase, Web of Science, Scopus, and the EMF-Portal. The reference lists of included studies and retrieved review articles will be manually searched.

**Study eligibility and criteria:**

We will include randomized human experimental studies that assess the effects of RF-EMFs on cognitive performance compared to no exposure or lower exposure. We will include peer-reviewed articles of any publication date in any language that report primary data.

**Data extraction and analysis:**

Data will be extracted according to a pre-defined set of forms developed and piloted by the review author team. To assess the risk of bias, we will apply the Rating Tool for Human and Animal Studies developed by NTP/OHAT, supplemented with additional questions relevant for cross-over studies. Where sufficiently similar studies are identified (e.g. the heterogeneity concerning population, exposure and outcome is low and the studies can be combined), we will conduct random-effects meta-analysis; otherwise, we will conduct a narrative synthesis.

**Assessment of certainty of evidence:**

The certainty of evidence for each identified outcome will be assessed according to Grading of Recommendations Assessment, Development, and Evaluation (GRADE).

Performing the review according to this protocol will allow the identification of possible effects of RF-EMFs on cognitive performance in humans.

The protocol has been registered in PROSPERO, an open-source protocol registration system, to foster transparency.

## 1 Introduction

### 1.1 Background

The technological applications of radiofrequency electromagnetic fields (RF-EMFs; frequencies ranging from 100 kHz to 300 GHz) have steadily increased since the 1950s. RF-EMFs are used in medicine (e.g. magnetic resonance imaging, diathermy, radiofrequency ablation), industry (e.g. heating and welding), domestic appliances (e.g. baby monitor, Wi-Fi), security and navigation (e.g. radar and radio frequency identification, RFID) and especially in telecommunications (e.g. radio and TV broadcasting, mobile telephony). These developments mean that large parts of the global population are already exposed to RF-EMFs and will be even more exposed in the future. Concern has been raised regarding public health consequences from RF-EMFs, and it is therefore crucial to identify possible hazards (adverse health outcomes) to support decision-makers and the general public. The World Health Organization (WHO) has an ongoing project to assess potential health effects of exposure to RF-EMFs in the general and working population. To prioritize potential adverse health outcomes from exposure to these fields, the WHO conducted a broad international survey amongst RF experts in 2018 (Verbeek et al., 2021). Six major topics were identified (cancer, adverse reproductive outcomes, cognitive impairment, symptoms, oxidative stress, and heat related effects) for which the WHO has commissioned systematic reviews of observational and experimental studies to analyze and synthesize the available evidence. In the current paper, we present the protocol for a systematic review of human experimental studies of short-term exposure to RF-EMFs and cognitive performance in humans. Observational studies on humans considering long-term exposure are the subject of another systematic review.

### 1.2 Description of the condition

The condition of interest in relation to RF-EMF exposure for this systematic review will be cognitive performance. Cognition is the set of mental processes fundamental for acquiring knowledge and understanding the world by means of thoughts, experiences, and the basic senses (Eysenck, 2012). Thus, it comprises several aspects of mental processes or functions such as attention, mental representation of knowledge, memory, reasoning, judgment, problem solving and language. The processes and functions can be assessed by applying visual, auditory or motor tasks. There are different models to describe different domains and subdomains of cognitive function and performance (e.g., Lezak et al., 2012, American Psychiatric Association, 2013). Most of the models include the domains “memory”, “attention” and “executive functions”, whereas some further domains vary, like e.g. “perceptual-motor function” or “information processing”. Irrespective of the proposed model, most of the cognitive tasks do not reflect one single domain, but involve multiple domains, like e.g. “attention” and “memory” when performing a short-term memory task. In some of the reviews and/or meta-analyses on possible effects of RF-EMFs on cognition, measures of cognitive performance have been categorized according to the cognitive tests that were applied (e.g., Trail Making Test, Choice Reaction Time Task) in the reviewed studies (e.g. Barth et al. 2008; 2012; Kwon and Hämäläinen, 2011). Other authors have categorized cognitive outcome parameter with respect to “cognitive performance functions” or “cognitive performance measures” like “reaction times” and “accuracy of performance” (e.g. Martens, 2005, Mortazavi et al., 2014, Regel and Achermann, 2011). Some authors classified cognitive performance into terms of “memory”, “attention”, “concentration”, or “working memory” (e.g. Hossmann and Hermann, 2003; van Rongen, 2009; Rubin et al., 2011). These different approaches to categorize cognitive performance reflect that there is no single, valid, agreed-upon model of cognitive domains. Furthermore, in RF-EMF research, not all cognitive domains may have been addressed and some tests might fit to more than one domain.

For this review, the classification system of Lezak et al. (2012) is most relevant, as it is one of the most comprehensive and internationally recognized textbooks on neuropsychological assessment. It provides a compendium of more than 750 different tests and assessment techniques and allows the grouping of cognitive performance tests into the following cognitive domains:

(1) Orientation and attention (e.g., attentional capacity, working memory, divided attention)
(2) Perception (e.g., auditory discrimination, face recognition)
(3) Memory (e.g., visual memory, word span)
(4) Verbal functions and language skills (e.g., verbal fluency, reading)
(5) Construction and motor performance (e.g., drawing, finger tapping)
(6) Concept formation and reasoning (e.g., card sorting, calculation)
(7) Executive functions (e.g., planning, decision making)

### 1.3 Description of the exposure

Radiofrequency electromagnetic fields (RF-EMFs) are defined as fields with frequencies ranging from 100 kHz to 300 GHz. Such fields are generated by a large number of devices as well as infrastructure both in the living environment and in workplaces.

Dependent on the geometry (e.g. size) and electrical characteristics of the source (e.g. frequency), and the distance and position of the exposed person to the source, exposure can occur almost uniformly over the entire body or more or less localized to a particular body region.

Additionally, RF-EMF exposure can occur in the far field or in the near field of the source. Due to different physical characteristics of RF-EMF in the near field compared to far field, also the coupling of the external RF-EMF into the body may be significantly different.. In the far field region, for the electric field vector **E** (measured in V/m), magnetic field vector **H** (measured in A/m) and the vector of the power flux density **S (**measured in W/m^2^) the simple relation |**E**|*|**H**|=|**S**| is valid and therefore, it is sufficient to quantify either **E** or **H** or **S** for a full characterization of the incident RF-EMF (Durney et al 1986).

In the near field region, the relationship and orientation between **E** and **H** vary significantly and are highly dependent on the detailed geometric and electric characteristics of the source. Moreover, if the source is operated very close to the body, i.e. the body is in the so called reactive near field, the exposed body itself may strongly interact with the source and thereby influence the radiation characteristics of the source.

The distance to the source, from which radiative near field or far field conditions can be assumed, depends on the geometric details (overall size) of the source and the frequency (or wavelength).

Overall, this leaves open a wide variety of possible exposure conditions in practice (i.e. whole body vs. localized exposure and far field vs. near field exposure).

In all cases, part of the RF power emitted by the source is absorbed into the body of the exposed person and may interact biologically with the body tissues. In general, the quantification of the absorbed power inside the different body tissues is highly complex and requires sophisticated numerical resources (e.g. anatomical body models and computer simulation capabilities). Experimentally, the absorbed RF power inside the body can only be determined by measurements in highly simplified body models (i.e. phantoms).

For uniform whole-body exposure under far field conditions the distribution of absorption inside the body has been analyzed extensively, and based on these results, the relationship between the incident electric field strength **E** (or alternatively the incident magnetic field strength **H**, or the incident power flux density **S**) and the specific absorption rate (SAR, measured in W/kg) inside the body is well established (ICNIRP, 2020, Bakker et al., 2011). Moreover, in the frequency range above 6 GHz absorption mainly takes place superficially (i.e. inside the skin). Under far field conditions, the incident **E**, **H**, or **S** can therefore be seen as a reasonable proxy for the RF-EMF absorption inside the human body for a given body size, body posture and body orientation with respect to the incident field.

In contrast, for exposure by RF-EMF sources operating close to the body, i.e. under near field conditions, in general a simple relationship between an easily measurable quantity (e.g. **E**, **H**, and **S**) and the absorption inside the body does not exist. In such cases, an accurate quantification of RF-EMF absorption requires detailed numerical computations of SAR inside the tissues for the particular situation, taking into account all relevant parameters of the RF-EMF source, the exposed part of the body and the specific geometrical relationship (distance, orientation) between the RF-EMF source and the exposed part of the body. This is particularly true for the case where the RF source (antenna) is operated directly at the ear. In such cases, the spatial peak average SAR may only be a poor proxy for brain exposure, when all head tissues (i.e. including the pinna) are taken into account for averaging (Keshvari et al. 2016). This is because the type of the antenna and its specific location with respect to the ear (pinna) may substantially affect the SAR distribution. There may be situations, for example, in which large amounts of the transmitted RF power may be absorbed in the pinna, and the subsequent absorption in the brain may therefore be significantly lower than in other situations with a similar spatial peak average SAR (including all head tissues). Therefore, a brain region specific SAR analysis would be favourable in such cases.

In addition to a widely varying exposure pattern, with respect to the spatial distribution of RF-EMF absorption inside the body, the time course of exposure intensity may also vary widely. In practice, depending on the particular exposure situation/application the exposure may occur continuously at constant intensity over long periods of time (e.g. from TV broadcast transmitters) or highly fluctuating due to complex modulation and power control schemes of modern mobile communication equipment (Kühn and Kuster 2012). In the latter case, exposure intensity may vary over several orders of magnitude within less than a microsecond.

If exposure is accumulated over time, the resulting cumulative dose can be expressed either in terms of energy density (J/m^2^) or specific absorbed energy (J/kg). These dose metrics have been established in epidemiology research to account for the huge variety of exposure durations experienced from different source types. However, for controlled experimental studies exposure metrics are commonly used (Roser et al. 2015; Lauer et al., 2013; Liorni Liorni et al., 2020).

### 1.4 Rationale for a systematic review

Investigation of potential effects of RF-EMFs on cognitive performance is mainly motivated by the RF-EMF exposure of the brain during mobile phone calls. Even small changes in performance measures such as accuracy, reaction time or performance speed may have a significant impact in certain cases due to the widespread use of RF-EMF transmitting devices among workers and in the general population.

Acute head-internal exposures from base stations, small cells or Wi-Fi routers are considerably lower than the potential exposure from a mobile phone operated close to the head. Nevertheless, there are health complaints from the general population, including cognition and concentration problems by residents living close to a base station or having Wi-Fi router in their homes (Röösli et al., 2004, Slottje et al., 2017). A subgroup of people considers themselves to be especially vulnerable and suffering from “electro-hypersensitivity” (idiopathic environmental intolerance attributed to EMF, IEI-EMF), although there is no scientific proof that the relationship is causal (Röösli, 2008, Rubinet al., 2011). It is also important to identify particularly sensitive population groups, if they exist. Children and adolescents could be at a higher risk, as they may be more sensitive and their lifetime exposure will start earlier and last longer than in the previous generation; although the actual state of knowledge is controversial (Wiedemann and Schütz, 2011, Loughranet al., 2013, Foerster et al., 2018). Elderly persons or individuals already suffering from various diseases could also be especially vulnerable, if RF-EMFs further impair their health condition (Redmayne and Johansson 2015).

Mechanisms for potential effects on cognition are unknown. Concerning exposure levels resulting from mobile phones, thermal effects have been suggested (Danker-Hopfe et al., 2016, Loughran et al., 2019). The RF exposure could improve or impair cognitive performance, depending on the cognitive challenge, the type of exposure, the outcome domain as well as other factors.

A high number of studies testing immediate effects of exposures have been conducted with volunteer participants, with varying reports of both improved and reduced performance, as well as statistically non-significant effects (Regel et al., 2007, Sauter et al., 2011, Sauter et al., 2015, Verrender et al., 2016, Zentai et al., 2015). Several meta-analyses (Barth et al. 2008, 2012; Valentini et al. 2010) and (systematic) reviews (Hamblin and Wood, 2002, Hossmann and Hermann, 2003, Martens, 2005, Cook et al., 2002, Cook et al., 2006, D'Andrea et al., 2003a, D'Andrea et al., 2003b, Sienkiewicz et al., 2005, van Rongen et al., 2009, Marino and Carrubba, 2009, Regel and Achermann, 2011, Rubinet al., 2011; Kwon and Hämäläinen, 2011; Wiedemann and Schütz, 2011, Mortazavi et al., 2014, Zhang et al., 2017, Curcio, 2018) on possible effects of RF-EMFs have been published to date, but the last meta-analysis had been published in 2012 (Barth et al. 2012). Furthermore, reviews, that included studies from the last ten years considered either one cognitive domain only (Curcio 2018), did not consider performance outcomes (Zhang et al. 2017), looked at possible beneficial effects only (Mortazavi et al., 2014) or considered only studies on children (Sienkiewicz et al., 2005, Wiedemann and Schütz, 2011). An up-to-date systematic review is needed to explore whether RF-EMF exposure may have an adverse effect on different measures of cognitive performance.

## 2 Objectives

The systematic review will provide a comprehensive analysis of the following PECO (Population, Exposure, Comparator, and Outcome) question:

To assess the immediate effects of exposure to RF-EMFs (E) on cognitive performance (O) in humans (P) compared to no or lower exposure level (C), and, if possible, to evaluate the dose–response relationship^1^ between exposure and outcome.

We define immediate effects as outcomes that have occurred during or immediately (within a few hours) after exposure within the given experimental time parameters and settings. Since possible mechanisms on the human brain and cognitive performance are not clear, we will not set a minimum duration of the exposure. We will consider the possible carry-over effects that might occur within our risk of bias assessment and used as orientation the recommendations for the conduction of cognitive performance measures in bioelectromagnetic research of Regel and Achermann (2011), who suggested that carry-over-effects might disappear after 24hrs.

## 3 Methods

The methodology of this systematic review is based on the WHO handbook for Guideline Development (WHO 2014) and will be reported in accordance with the Preferred Reporting Items for Systematic Reviews and Meta-Analyses (PRISMA) guidelines (Moher et al. 2009).

### 3.1 Eligibility criteria

**Fig. 1:**
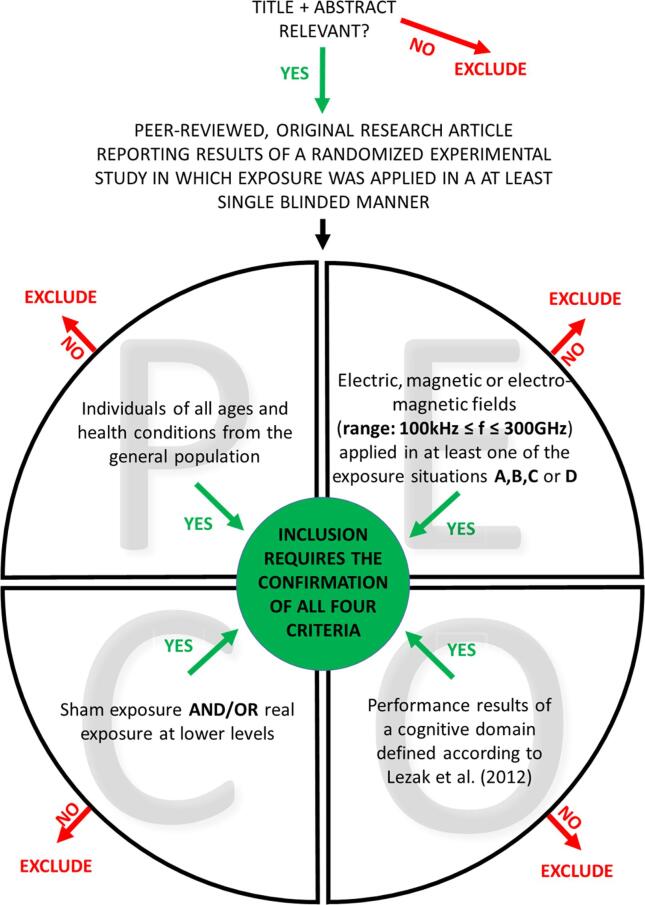
Graphical summary of the study selection process. The PECO questions can be answered in any order. Any single “no” response will lead to the exclusion of the study. For inclusion, all questions have to be answered with “yes”.

The eligibility criteria with regard to the PECO are described in detail below and summarized in Fig. 1.

#### 3.1.1 Types of populations

We will be non-restrictive with regard to the population, and will include healthy and non-healthy individuals of all ages from general and occupational populations; thus, relevant studies may be conducted in the general or occupational population of all ages comprising adults, adolescents and children of any health condition, healthy volunteers and patients, as well as individuals with IEI-EMF.

#### 3.1.2 Types of exposures

We will include all identified studies that have applied electric, magnetic or electromagnetic fields in the frequency range of 100 kHz to 300 GHz and that have reported exposure using at least one of the situations listed below:

A) **body/tissue internal exposure metrics** measured or calculated for the particular conditions of the experiment,

• *SAR* [*expressed in* W/kg *or equivalent units*]
• *SA* [*expressed in* J/kg *or equivalent units*]
• *induced electric field strength, [expressed in V/m or equivalent units]*
• *internal magnetic field strength [expressed in A/m or equivalent units]*

(For exposure applied as pure or predominantly magnetic fields in the lower frequency range *the external magnetic field strength at body position* is considered a sufficient surrogate for the *tissue internal magnetic field,* as long as the penetration depth is high compared to the dimension of the body)

B) ***body*/*tissue internal exposure metrics*** *describing superficial absorption at frequencies above 6 GHz measured or calculated for the conditions of the experiment as follows,*

• *incident power flux density* [expressed in W/m^2^ or equivalent units]
• *incident energy density* [expressed in J/m^2^ or equivalent units]
• *transmitted (absorbed) power flux density* [expressed in W/m^2^ or equivalent units]
• *transmitted (absorbed) energy density* [expressed in J/m^2^ or equivalent units],

C) ***body*/*tissue external exposure metrics***

• *external electric field strength* [V/m] (E > 1 V/m or E > √10*background level in unshielded environment, otherwise no restriction)
• *external magnetic field strength* [mA/m] (H > 2.7 mA/m or H > √10*background level in unshielded environment, otherwise no restriction)
• *incident power flux density*, [mW/m^2^] (PD > 2.5 mW/m^2^ or PD > 10*background level in unshielded environment, otherwise no restriction)

We will only include studies reporting external metrics according to situation C if (i) either of these exposure metrics was measured or calculated at the location of the exposed body/tissue in the approximate far-field of the field source, and (ii) the exposure level is at least a factor of 10 (power flux density) or √10 (field strength) above background level^2^. In the case where a study reports no specific background exposure level in the laboratory, we will assume a value of 0.25 mW/m^2^ (corresponding to 0.3 V/m and 0.9 mA/m, respectively) as the background exposure level. This results in an inclusion threshold of PD = 2.5 mW/m^2^, E = 1 V/m, or H = 2.7 mA/m).

D) ***mobile phones or other RF-generating devices as source of exposure without reporting of metrics under A, B or C***

1. With output controlled by appropriate software or hardware operated close to the body/tissue

We will include studies that applied exposure with an output power controlled by hardware or software, provided that the output power and the distance to the body/tissue are reported, enabling inference of the exposure.

2. In GSM mode with an active call operated close to the body/tissue

An exposure applied as the field generated by a mobile phone in GSM mode with an active call operated at distances equal or less than 3 cm from the body/tissue can be expected to generate a temporal peak SAR in the range of 0.01 to 100 W/kg, which is at least a factor 100 above the average background level. We will include these studies only if the active call was maintained throughout the experiment and the comparison was a similar phone switched off.

We will exclude studies that

• have applied exposure signals with >10% of the total signal energy outside the considered frequency range 100 kHz – 300 GHz (e.g. pulsed fields, non-sinusoidal fields with dominant frequencies less than 100 kHz).
• have applied exposure with a mobile phone not in GSM mode or not in active call or not controlled by hardware or software, because of the extremely small exposure contrast generated which we do not consider relevant.

#### 3.1.3 Types of comparators

We will include studies with a control condition comprising either sham exposure, no exposure beyond background exposure level (which can be assumed negligibly low), or a RF-EMF exposure at a lower level.

#### 3.1.4 Types of outcomes

We will include studies that have assessed the following cognitive domains according to Lezak et al. (2012):

(1) Orientation and attention
(2) Perception
(3) Memory
(4) Verbal functions and language skills
(5) Construction and motor performance
(6) Concept formation and reasoning
(7) Executive functions

The domains can be measured in terms of accuracy (e.g. correct, false, missed responses) and/or speed of performance (e.g. reaction time). Beyond those tests described by Lezak et al., 2012, Fulda and Schulz, 2001 provide a list of further tests and group these according to Lezak’s taxonomy. If a certain test is not indexed in any of the two sources, it will be grouped according to the primarily required measure of cognitive performance.

#### 3.1.5 Types of studies

##### 3.1.5.1 Inclusion criteria

We will include parallel-group or cross-over randomized experimental studies with at least two RF-EMF exposure conditions (including sham or control) in a laboratory or at other locations (e.g. working place, home) under controlled conditions, in which at least the participants were blind to the exposure condition (i.e. whether it is the exposure or the control condition).

We will only consider these specific study designs as evidence for an effect because there is a considerable risk of confounding and performance bias if the exposure sequence is not randomized and/or participants are not blinded for the type of exposure.

##### 3.1.5.2 Years considered

We will consider studies published in any year.

##### 3.1.5.3 Publication language

We will consider studies written in any language. For languages not covered by the author team, we will first use Google translate, and if needed we will seek help from a native speaker for inclusion and data extraction. The WHO will endeavor to assist the team in finding help to assess inclusion and data extraction if necessary.

##### 3.1.5.4 Publication types

We will consider peer-reviewed articles that report primary data.

##### 3.1.5.5 Exclusion criteria

We will exclude non-randomized studies and studies where participants were not blinded to the exposure because confounding and selection bias make it difficult to establish causal effects and render studies at a potentially critical risk of bias.

We will exclude reviews, statements, reports, opinion papers, comments, editorial, conference abstracts or proceedings.

### 3.2 Information source and search strategy

The search strategy combines controlled vocabulary and keyword terms related to the exposures and outcomes in the PECO statement above. The terms were adjusted to achieve a balance of precision and comprehensiveness in results. A pre-determined set of target articles, which we knew should be included, was used to iteratively evaluate the search strategy.

The following databases will be searched: PubMed, Embase, Scopus, and Web of Science. We will also search the EMF Portal, a dedicated database of the scientific literature on health effects of exposure to electromagnetic fields (https://www.emf-portal.org/en). The search strategy for each database is recorded in appendix A1-A5.

The search results will be imported into the reference management software EndNote®, and duplicates will be removed.

This search will be supplemented by checks of the reference lists of previous reviews, as identified by the literature search, and by checks of reference lists in eligible publications.

### 3.3 Study selection

Records and data will be managed using the Covidence web application for systematic reviews. For study selection, search results will be imported into Covidence from EndNote. In a first step, two reviewers will independently check the relevance of the identified papers based on titles and abstracts. At this stage, we will exclude records that are clearly not relevant and will certainly not fulfil one or more of the inclusion criteria listed above. This will result in a list of references for which in a second step, again two reviewers will independently assess the inclusion and exclusion criteria based on the full text of the article. The study selection process is summarized in Fig. 1. The reasons for exclusion of full-text articles will be recorded in Covidence. In all steps, any disagreement between the two reviewers will be resolved by discussion. If no consensus can be reached, a third reviewer will be consulted. If findings from a study are described in more than one article, we will consider all these papers as one study only. If one paper reports several independent experiments, they will be considered as separate studies. This study selection process will result in a list of included studies.

We will document the selection process in a study flow diagram according to the PRISMA reporting guidelines (Liberati et al., 2009).

### 3.4 Data extraction

For each study included in the review, a standard set of details will be extracted from the relevant publication(s) using Covidence.

One reviewer will extract and record the relevant features of each eligible study. A second reviewer will check the extracted study information against the accompanying article(s) for completeness and accuracy as a quality control measure. When studies conducted by the authors of this review are included, we will make sure that these authors do not extract data from their own studies.

A data extraction form based on the extraction forms predefined in Covidence was developed by the reviewers for the purpose of this review; it was tested independently by two reviewers on three selected studies, subsequently adapted, and agreed upon by all review authors. The data extracted from each study are listed in Appendix B.

Dealing with missing data:

For any studies for which missing data preclude the determination of eligibility, assessment of risk of bias or inclusion in the data synthesis, we will request information from the corresponding author via email. If no response is received, we will follow up twice within approximately four weeks. If there is still no response, we will consider the data as missing. We will document any correspondence with the authors in an appendix. If information necessary for data analysis is missing from the articles, we will try to calculate these from other information that is reported.

### 3.5 Risk of bias assessment

#### 3.5.1 Risk of bias in studies

We will apply the Risk of Bias (ROB) Rating Tool for Human and Animal Studies developed by the NTP Office of Health Assessment and Translation (OHAT) (NTP-OHAT (National Toxicology Program, Office of Health Assessment and Translation), 2015, Rooney et al., 2014, Rooney et al., 2016) to assess the risk of bias of included studies. This tool has various forms for different study designs; we will primarily use the questions designed for human clinical trials. The OHAT questions for human clinical trials, however, are designed primarily for parallel group studies. To make sure that cross-over studies, which we expect to identify, are adequately assessed, we have added three further questions. One of these questions is from the OHAT questions for experimental animal studies, and the other two are from the Cochrane RoB 2.0 tool (Higgins 2016). These three additional questions are indicated below. We will consider the following domains and questions:

**Selection bias**

1. Was administered exposure level adequately randomized?

2. Was allocation to study groups^3^ adequately concealed?

3. For cross-over studies: Is an equal proportion of participants allocated to any study group to prevent a period effect^4^? (**Note**: we adapted this question from RoB 2.0, questions 1.4 and 1.5)

**Performance bias**

4. Were experimental conditions identical across study groups? (**Note**: we adapted this question from OHAT tool for experimental animal studies.)

5. Were the research personnel and human subjects blinded to the exposure situation during the study?

6. For cross-over studies: Was there sufficient time for any carry-over effects in cross-over studies to have disappeared before outcome assessment in the second period? (**Note**: we adapted this question from RoB 2.0, question 2.5)

Attrition / Exclusion bias

Were outcome data complete without attrition or exclusion from analysis?

**Detection bias**

8. Can we be confident in the exposure characterization and is the exposure contrast sufficient?

9. Can we be confident in the outcome assessment?

**Selective reporting bias**

10. Were all measured outcomes reported?

Each item, in the form of a specific question, is rated with one of four options: (i) definitely low risk of bias, (ii) probably low risk of bias, (iii) probably high risk of bias or no information/unclear risk of bias and (iv) definitely high risk of bias. Considerations and guidance for rating the single items are given in Genaidy et al., 2007, Higgins, 2011. Where contacting authors cannot provide missing data, as per the guidance for the OHAT tool, the particular question will be assigned “probably high risk of bias”.

The RoB will be assessed independently by two reviewers using the forms pre-defined in Covidence. Any disagreement will be resolved by discussion. If necessary, a third reviewer will be consulted. When studies conducted by the authors of this review are included, we will make sure that these authors do not assess the RoB of their own studies.

#### 3.5.2 Assessment of reporting bias

To assess publication bias, where data allow it (i.e. where meta-analyses are conducted) we will create and visually examine funnel plots. In addition, we will statistically assess potential funnel plot asymmetry with the Egger’s test (small study effects).

### 3.6 Synthesis of results

As we are considering a range of outcomes, and based on preliminary scoping and our knowledge of the evidence base, we expect that included studies will have assessed many outcomes in a range of forms. We will compile all effect estimates from included studies and consider how to best utilize these measures across studies. We expect many studies to report reaction times and the proportion of correct responses; other continuous measures of cognitive performance may be expressed as mean differences (MDs) or standard mean differences (SMDs), such as Cohen’s d or Hedge’s g values. Dichotomous measures may be expressed as odds ratios (OR) or risk ratios (RR). Where studies report another measure, if possible, we will convert this into one of these measures for further analysis. For all other studies, we will consider outcomes as they are reported in the study.

Based on our preliminary knowledge of the evidence base, we expect substantial heterogeneity with regard to the assessed population, exposure, outcome and study methodology. RF-EMF exposure and cognitive tests, for example, may vary widely across studies, and small differences in content or methodology could mean that statistical combination of effects across studies is not appropriate. Nevertheless, where included studies for a given outcome are sufficiently similar across PECO aspects and study methodology, we will conduct meta-analyses; due to the expected heterogeneity, any analyses we conduct will use random effects meta-analysis.

Regarding the population we will include individuals of all ages into the main analyses; however, we will conduct separate analyses for healthy populations and patient populations. Likewise, we will conduct separate analyses for individuals with and without IEI-EMF.

Studies that meet exposure eligibility criteria and report external or internal exposure metrics according to 3.1.2. A, B or C are considered as sufficiently similar to be combined in the main analyses. We will consider studies referring to mobile phone or other RF generating devices without reporting of metrics under A, B or C (3.2.1. D) separately, because there is variation and/or uncertainty in exposure levels. We will consider all exposure levels within each group as sufficiently similar to be combined in the main analyses.

With regard to the comparator, we will consider both sham and no exposure (if properly blinded) as sufficiently similar to be combined in the main analyses.

Regarding the outcomes, each of the seven cognitive outcome domains listed above will be analyzed separately.

For studies from which the effect measure could not be converted into a common standard metric for the meta-analysis, or where no meta-analysis is possible or appropriate, we will synthesize findings narratively, assessing each outcome separately. We will supplement this narrative synthesis with structured tables, and where possible we will also summarize effect estimates for each outcome using forest plots without a summary statistic.

### 3.7 Additional analyses

#### 3.7.1 Exposure-response analysis

If applicable, we will also perform an exposure–response analysis by applying exposure level as a continuous variable. We will only include studies in this analysis if the method for determining the exposure level is provided and is rated to be probably or definitively at low risk of bias.

Concerning studies with localized exposure of the head, the local SAR inside the brain will be used as the relevant exposure metric for the exposure–response analysis. Ideally, a brain region-specific exposure analysis would be provided in the included studies and the local SAR (spatial peak 1 g- or 10 g-averaged SAR and/or mass averaged SAR) inside the brain region of interest can be used for the exposure–response analysis. However, we expect that most studies will only provide information about the spatial peak SAR in the head.

Therefore, the spatial peak SAR averaged over 10 g (SAR_10g_) of brain tissue or head tissue (excluding ear tissue) is also assumed an appropriate exposure metric. When maximum local SAR_1g_ is provided, conversion to SAR_10g_ for the same region can be done:

• SAR_10g_ = 0.6 *SAR_1g_ (Hirata et al, 2006)

Concerning studies in which the whole body or a large part of the body has been exposed and far field conditions can be assumed, we will also carry out an exposure–response analysis based on appropriate exposure metrics. This will be done in order to take into account that a more “general stress” by the total body “load” of the exposure might also be of relevance.

In such cases, the following exposure metrics are considered appropriate:

• The ratio between whole-body average SAR and the ICNIRP (2020) basic restriction value of 0.08 W/kg for the whole-body average SAR for the general public.
• The ratio between the incident power density and the ICNIRP (2020) reference level for incident power density.
• The square of the ratio between the incident electric/magnetic field strength and the ICNIRP (2020) reference level for incident electric/magnetic field strength.

For frequencies above 300 MHz we will regard these quantities as equivalent for all polarisations, because at these frequencies the effect of polarization is insignificant, except for k-polarization (Durney et al., 1986, Hirata et al., 2009, Kühn et al., 2009, Vermeeren et al., 2008). At lower frequencies, i.e. closer to or at the whole-body resonance frequencies, the effect of polarization will be significant. For exposures with k-polarisation or at frequencies below 300 MHz we will only use the ratio between whole-body average and SAR 0.08 W/kg as the exposure metric. If the whole-body average SAR is not provided, the experiment will not be included in the exposure–response analysis.

The exposure–response analyses will be carried out for temporal average values as well as for temporal peak values of the respective exposure metric. In case only temporal average values of SAR are provided, corresponding temporal peak values will also be obtained based on the temporal characteristics of the exposure signal, and vice versa.

#### 3.7.2 Heterogeneity and subgroup analyses

Where meta-analyses are possible, we will quantify the statistical heterogeneity between studies using the tau-square measure and 80% prediction intervals (PI) to estimate the 80% interval in which the true effect measure will lie (Chiolero et al., 2012). Severe heterogeneity will be assumed when PIs include the null effect, and at the same time, the 80% PIs are larger than the 95% confidence intervals (CI) by a factor of two.

Statistical heterogeneity is the result of clinical heterogeneity (e.g. between-study differences related to the populations, exposures, comparators or outcomes) and/or methodological heterogeneity (e.g., between-study differences in how exposures are applied, or outcomes are measured). Related to clinical heterogeneity, for example, the effect on cognitive performance might differ between men and women or different age groups. Furthermore, the effect might depend on the frequency and modulations characteristics of the exposure. Specifically, we expect that the following aspects may lead to heterogeneity:

Different sub-populations:

• Sex
  – females
  – males
• Age groups
  – children and adolescents
  – adults
  – elderly

Different exposure types:

• Signals with different modulation
  – unmodulated (continuous wave)
  – TETRA-like
  – GSM-like
  – UMTS-like
  – WLAN-like
  – LTE-like
  – additional communication standards if used in the included studies
• Time course of exposure
  – Signals with intermittent pattern, i.e., if one of the above signal types is applied according to a specific time pattern, e.g. several seconds/minutes ON / several seconds/minutes OFF
  – Signals without intermittent pattern
• Exposure source
  – far field source
  – near field source
• Clusters of specific non-overlapping local exposure levels (max. 10 g average SAR)
  – considerably below the limit of 2 W/kg for local exposure of the general public
  – close to the limit of 2 W/kg for local exposure of the general public
  – considerably above 2 W/kg, close to the occupational exposure limit
• Clusters of specific non-overlapping whole body exposure levels (whole body average SAR)
  – considerably below the limit of 0.08 W/kg for whole body exposure of the general public
  – close to the limit of 0.08 W/kg for whole body exposure of the general public
  – considerably above 0.08 W/kg, close to the occupational exposure limit
• **Different frequencies:**
  – below 1 GHz
  – 1 – 3 GHz
  – above 3 GHz
• **Blinding:**
  – single blinded studies
  – double blinded studies

Different funding sources:

– government
– industry
– non-government organization (NGO)
– mixed (firewall prevents influence of industry))
– unknown

We will conduct subgroup analyses stratified for the above-listed aspects to explore the extent to which between-study heterogeneity may moderate the results of our meta-analyses, where data from included studies allow it. Where no meta-analysis is possible, we will highlight heterogeneity narratively in tables and/or figures based on the above-listed aspects. These narrative assessments will not allow us to derive conclusions but could point to aspects potentially modifying the effectiveness of different measures.

#### 3.7.3 Sensitivity analyses

We will assess the robustness of assumptions made in the application of risk of bias tool, by comparing the results from studies considered at high risk of bias versus the results from studies at low risk of bias.

### 3.8 Certainty of evidence assessment

We will assess the certainty of evidence of each body of evidence according to the “Grading of Recommendations Assessment, Development, and Evaluation (GRADE) approach" (Guyatt et al. 2011a). A body of evidence will comprise studies deemed similar enough with regard to population and exposure quality to be analyzed together (we consider all exposure levels and comparators as sufficiently similar) and assessing a specific outcome domain, as described above. If it is not possible to pool the data across studies (i.e. perform a meta-analysis), the GRADE approach will be applied narratively as described in a recent guidance (Murad et al 2017).

In the GRADE approach, randomized controlled experimental studies start as high-certainty evidence supporting estimates of exposure effects. Five factors may lead to rating down the certainty of evidence: study limitations, indirectness of evidence, inconsistency of results, imprecision and publication bias.

• Study limitations (possibility for rating down: one or two levels): the certainty of evidence will be rated down if the respective studies are at substantial risk of bias. The risk of bias will be judged across studies for an individual outcome. There will be no specific threshold for determining when to rate down, but we will consider the impact that bias could have on the summarized effect.
• Indirectness of evidence (possibility for rating down: one or two levels): the certainty of evidence will be rated down if the evidence does not exactly reflect the PECO, e. g it is based on indirect measures of the outcome. Given that we have defined tight eligibility criteria, however, we do not expect to identify any indirect bodies of evidence.
• Inconsistency of results (possibility for rating down: one or two levels): the certainty of evidence will be rated down if considerable differences in the magnitude or direction of the effect are observed between studies.
• Imprecision (possibility for rating down: one or two levels): the certainty of evidence will be rated down if the 95% CI is wide and includes a relative risk of 1.0, or if the total number of individuals required for an adequately powered individual study is not reached (termed the ‘‘optimal information size’’, estimated according to Guyatt et al 2011b).
• Publication bias (possibility for rating down: one level): the certainty of evidence will be rated down when the body of evidence consists mainly of positive studies with small sample sizes or when studies or if the funnel plot upon visual inspection shows that small studies with non-harmful effects are missing. We will then use Egger’s test to confirm this.

Ultimately, after the application of each of these factors, the certainty of evidence for each body of evidence falls into one of four categories: high, moderate, low and very low certainty. Based on the certainty of evidence, we will conclude if RF-EMF exposure affects cognitive performance.

## 4 Conclusion and future perspectives

The conduct of the systematic review according to this protocol will allow for the assessment of possible adverse health effects of RF-EMFs on cognitive performance in humans, and where possible an evaluation of the associated dose–response relationship. The results of several systematic reviews commissioned by the WHO will allow an improved risk assessment and risk communication by decision makers, related to the public use of mobile communications.

## 5 Financial support

This project is funded by the World Health Organization.

## 6 Registration

PROSPERO CRD42021236168.

## CRediT authorship contribution statement

**Blanka Pophof:** Project administration, Funding acquisition, Writing - review & editing. **Jacob Burns:** Methodology, Writing - original draft. **Heidi Danker-Hopfe:** Writing - review & editing. **Hans Dorn:** Writing - original draft. **Cornelia Egblomassé-Roidl:** . **Torsten Eggert:** Writing - original draft. **Kateryna Fuks:** . **Bernd Henschenmacher:** Writing - review & editing. **Jens Kuhne:** Writing - original draft. **Cornelia Sauter:** Writing - original draft. **Gernot Schmid:** Writing - original draft.

## Declaration of Competing Interest

The authors declare that they have no known competing financial interests or personal relationships that could have appeared to influence the work reported in this paper.

## Footnotes

1 Strictly speaking, exposure–response relationships are evaluated in this SR. Dose (e.g. applied/absorbed amount of compound of per unit mass (toxicology) or applied/absorbed energy per unit mass (ionizing radiation)) is a quantity that typically describes the accumulated exposure of the study object. According to current knowledge, EMF or EMF-induced effects do not accumulate in the body, which is why exposure–response relationships are of interest. By taking exposure time into account conversion of exposure–response relationships into dose–response relationships is possible.

2 Data from the literature (Jalilian et al., 2019) suggest RF E-fields of approximately 0.1 – 0.3 V/m (corresponding to PD of 25 – 250 µW/m2) as a reasonable range for typical indoor RF background exposure in a laboratory environment. From other studies (Christ et al., 2006a, Christ et al., 2006b; Schmid and Kuster, 2015), it can be derived that such background exposure levels may lead to SAR values inside superficial tissue layers and cell cultures in the order of several tens to several hundreds of µW/kg, and that a RF source with a low directivity antenna (e.g. mobile phone) operated within a few centimeters of the body may cause SAR values in superficial layers in the range of several tens to lower hundreds W/kg per watt transmit power. Hence, a mobile phone in GSM operating mode with an active call (peak transmit power in the range approx. 1 mW up to 2W) in close proximity to the body/tissue is expected to cause substantially higher (approx. factor 100–1000) temporal peak SAR than typical RF background exposure, even when the transmit power of the mobile phone (in GSM mode) was not controlled by the experiment, but autonomously controlled by the network. Despite the expected high variation of exposure in such cases, we will therefore also include studies that used a mobile phone in GSM mode with an active call at distances equal or less than 3 cm from the body/tissue as field source, provided that evidence is given that the active call was maintained throughout the experiment and that the control group (sham exposed group) was treated with the mobile phone switched off.

3 “Group” means subjects/persons/volunteers allocated to a fixed exposure condition or exposure condition sequence

4 “Period effects” are defined as systematic changes in outcome over time that cannot be attributed to the different exposure conditions. These include for example order or learning effects.
