# Supplementary material for: The effect of exposure to radiofrequency electromagnetic fields on cognitive performance in human experimental studies: A protocol for a systematic review
Source: Environ Int. 2021 Dec;157:106783. doi: 10.1016/j.envint.2021.106783 (PMC8485020; doi:10.1016/j.envint.2021.106783)
Supplement: Supplementary data 1 [file mmc1.docx]

**Pub Med**

(("Electromagnetic Radiation"[MeSH Terms:noexp] OR "electromagnetic wave*"[Text Word] OR "electromagnetic energ*"[Text Word] OR "electromagnetic radiation*"[Text Word] OR "Radio Waves"[MeSH Terms] OR "radio wave*"[Text Word] OR "radiowave*"[Text Word] OR "hertzian wave*"[Text Word] OR "high frequency wave*"[Text Word] OR "short wave*"[Text Word] OR "microwave field*"[Text Word] OR "microwave radiat*"[Text Word] OR "microwave expos*"[Text Word] OR "microwave irradiat*"[Text Word] OR "microwave range*"[Text Word] OR "micro wave radiat*"[Text Word] OR "mw field*"[Text Word] OR "mw radiat*"[Text Word] OR "mw expos*"[Text Word] OR "mw irradiat*"[Text Word] OR "mw range*"[Text Word] OR "m w range*"[Text Word] OR "ehf wave*"[Text Word] OR "ultrahigh frequency wave*"[Text Word] OR "UHF"[Text Word] OR "radiofrequenc*"[Text Word] OR "radio frequenc*"[Text Word] OR "rf wave*"[Text Word] OR "rf field*"[Text Word] OR "rf electric field*"[Text Word] OR "rf magnetic field*"[Text Word] OR "rf radiation*"[Text Word] OR "rf expos*"[Text Word] OR "RF EMF"[Text Word] OR "millimeter wave*"[Text Word] OR "Electromagnetic Fields"[MeSH Terms] OR "electromagnetic environment*"[Text Word] OR "electromagnetic field*"[Text Word] OR "electromagnetic phenomen*"[Text Word] OR "Electromagnetics"[Text Word] OR "Electromagnetism"[Text Word] OR "Radar"[MeSH Terms] OR "Radar"[Text Word] OR "Cell Phone"[MeSH Terms:noexp] OR "cell phone*"[Text Word] OR "cellphone*"[Text Word] OR "cellular phone*"[Text Word] OR "cellular telephone*"[Text Word] OR "mobile phone*"[Text Word] OR "mobile telephone*"[Text Word] OR "cordless phone*"[Text Word] OR "car phone*"[Text Word] OR "Smartphone"[MeSH Terms] OR "smartphone*"[Text Word] OR "smart phone*"[Text Word] OR "iphone*"[Text Word] OR "i phone*"[Text Word] OR "Android"[Text Word] OR "Cell Phone Use"[MeSH Terms] OR "Wireless Technology"[MeSH Terms] OR "wireless technolog*"[Text Word] OR "wireless communication*"[Text Word] OR "Wi-Fi"[Text Word] OR "Wifi"[Text Word] OR "specific absorption rate*"[Text Word] OR "W/kg"[Text Word] OR "global system for mobile communication*"[Text Word] OR "GSM"[Text Word] OR "digital cellular system*"[Text Word] OR "universal mobile telecommunication system*"[Text Word] OR "UMTS"[Text Word] OR "Code Division Multiple Access"[Text Word] OR "CDMA"[Text Word] OR "WCDMA"[Text Word] OR "WiMAX"[Text Word] OR "Bluetooth"[Text Word] OR "Terrestrial Trunked Radio"[Text Word] OR "TETRA"[Text Word] OR "digital enhanced cordless telecommunication*"[Text Word])

AND ("auditory task"[Text Word] OR "choice reaction"[Text Word] OR "clock monitoring"[Text Word] OR "contingent negative variation"[Text Word] OR "cognit*"[Text Word] OR "contingent negative variation"[Text Word] OR "critical flicker frequency"[Text Word] OR "critical fusion frequency"[Text Word] OR "decision making"[Text Word] OR "digit span"[Text Word] OR "discrimination task"[Text Word] OR "divided attention"[Text Word] OR "executive function"[Text Word] OR "executive function*"[Text Word] OR "information processing"[Text Word] OR "learning"[Text Word] OR "memory"[Text Word] OR "mental function*"[Text Word] OR "neural function"[Text Word] OR "neurocognit*"[Text Word] OR "neuropsycho*"[Text Word] OR "oddball"[Text Word] OR "order threshold"[Text Word] OR "performance accuracy"[Text Word] OR "performance speed"[Text Word] OR "psychomotor"[Text Word] OR "reaction time"[Text Word] OR "response time"[Text Word] OR "selective attention"[Text Word] OR "sentence verification"[Text Word] OR "simple reaction"[Text Word] OR "spatial compatibility"[Text Word] OR "spatial recognition"[Text Word] OR "speed of processing"[Text Word] OR "stroop"[Text Word] OR "sustained attention"[Text Word] OR "test battery"[Text Word] OR "trail making"[Text Word] OR "verbal fluency"[Text Word] OR "verbal item"[Text Word] OR "verbal performance"[Text Word] OR "verification task"[Text Word] OR "vigilance"[Text Word] OR "visual discrimination"[Text Word] OR "visual task"[Text Word] OR "word recall"[Text Word])

AND ("child*"[Text Word] OR "adolescen*"[Text Word] OR "adult*"[Text Word] OR "elderly"[Text Word] OR "human*"[Text Word] OR "individual*"[Text Word] OR "patient*"[Text Word] OR "participant*"[Text Word] OR "student*"[Text Word] OR "subject*"[Text Word] OR"volunteer*"[Text Word]))

AND ((journalarticle[Filter] OR review[Filter]) AND (humans[Filter]))
