# Supplementary material for: The effect of exposure to radiofrequency electromagnetic fields on cognitive performance in human experimental studies: A protocol for a systematic review
Source: Environ Int. 2021 Dec;157:106783. doi: 10.1016/j.envint.2021.106783 (PMC8485020; doi:10.1016/j.envint.2021.106783)
Supplement: Supplementary data 2 [file mmc2.docx]

**Embase**

('electromagnetic radiation'/exp OR 'electromagnetic radiation' OR 'electromagnetic energ*':ti,ab,kw OR 'electromagnetic radiation':ti,ab,kw OR 'electromagnetic wave*':ti,ab,kw OR 'radiofrequency radiation'/exp OR 'radiofrequency radiation' OR 'hertzian wave*':ti,ab,kw OR 'high frequency wave*':ti,ab,kw OR 'radio frequenc*':ti,ab,kw OR 'radio wave*':ti,ab,kw OR radiofrequenc*:ti,ab,kw OR radiowave*:ti,ab,kw OR 'rf electric field*':ti,ab,kw OR 'rf emf':ti,ab,kw OR 'rf exposure*':ti,ab,kw OR 'rf field*':ti,ab,kw OR 'rf magnetic field*':ti,ab,kw OR 'rf radiation':ti,ab,kw OR 'rf wave*':ti,ab,kw OR 'short wave*':ti,ab,kw OR 'microwave radiation'/exp OR 'microwave radiation' OR 'ehf wave*':ti,ab,kw OR 'm w field*':ti,ab,kw OR 'm w radiat*':ti,ab,kw OR 'm w range*':ti,ab,kw OR 'micro wave field*':ti,ab,kw OR 'micro wave radiat*':ti,ab,kw OR 'micro wave range*':ti,ab,kw OR 'microwave field*':ti,ab,kw OR 'microwave radiat*':ti,ab,kw OR 'microwave range*':ti,ab,kw OR 'mw field*':ti,ab,kw OR 'mw radiat*':ti,ab,kw OR 'mw range*':ti,ab,kw OR uhf:ti,ab,kw OR 'ultrahigh frequency wave*':ti,ab,kw OR 'microwave irradiation'/exp OR 'microwave irradiation' OR 'm w expos*':ti,ab,kw OR 'm w irradiat*':ti,ab,kw OR 'micro wave expos*':ti,ab,kw OR 'micro wave irradiat*':ti,ab,kw OR 'microwave expos*':ti,ab,kw OR 'microwave irradiat*':ti,ab,kw OR 'mw expos*':ti,ab,kw OR 'mw irradiat*':ti,ab,kw OR 'millimeter wave'/exp OR 'millimeter wave' OR 'millimeter wave*':ti,ab,kw OR 'electromagnetism'/exp OR 'electromagnetism' OR 'electromagnetic environment':ti,ab,kw OR 'electromagnetic field*':ti,ab,kw OR 'electromagnetic phenomena':ti,ab,kw OR electromagnetics:ti,ab,kw OR electromagnetism:ti,ab,kw OR radar:ti,ab,kw OR 'mobile phone'/exp OR 'mobile phone' OR android:ti,ab,kw OR 'car phone*':ti,ab,kw OR 'cell phone*':ti,ab,kw OR cellphone*:ti,ab,kw OR 'cellular phone*':ti,ab,kw OR 'cellular telephone*':ti,ab,kw OR 'cordless phone*':ti,ab,kw OR iphone*:ti,ab,kw OR 'i phone*':ti,ab,kw OR 'mobile phone*':ti,ab,kw OR 'mobile telephone*':ti,ab,kw OR 'smart phone*':ti,ab,kw OR smartphone*:ti,ab,kw OR 'cell phone use'/exp OR 'cell phone use' OR 'wireless communication'/exp OR 'wireless communication' OR 'wireless communication*':ti,ab,kw OR 'wireless technolog*':ti,ab,kw OR 'wi fi'/exp OR 'wi fi' OR 'wi fi':ti,ab,kw OR wifi:ti,ab,kw OR 'specific absorption rate'/exp OR 'specific absorption rate' OR 'specific absorption rate':ti,ab,kw OR 'w/kg':ti,ab,kw OR 'digital cellular system*':ti,ab,kw OR 'global system for mobile communication'/exp OR 'global system for mobile communication' OR 'global system for mobile communication*':ti,ab,kw OR gsm:ti,ab,kw OR 'total access communication system':ti,ab,kw OR umts:ti,ab,kw OR 'universal mobile telecommunication system*':ti,ab,kw OR cdma:ti,ab,kw OR 'code division multiple access':ti,ab,kw OR wcdma:ti,ab,kw OR 'bluetooth'/exp OR 'bluetooth' OR bluetooth:ti,ab,kw OR 'digital enhanced cordless telecommunication*':ti,ab,kw OR 'terrestrial trunked radio':ti,ab,kw OR tetra:ti,ab,kw OR wimax:ti,ab,kw)

AND (‘auditory task’:ti,ab,kw OR ‘choice reaction’:ti,ab,kw OR ‘clock monitoring’:ti,ab,kw OR ‘contingent negative variation’:ti,ab,kw OR ‘cognit*’:ti,ab,kw OR ‘contingent negative variation’:ti,ab,kw OR ‘critical flicker frequency’:ti,ab,kw OR ‘critical fusion frequency’:ti,ab,kw OR ‘decision making’:ti,ab,kw OR ‘digit span’:ti,ab,kw OR ‘discrimination task’:ti,ab,kw OR ‘divided attention’:ti,ab,kw OR ‘executive function*’:ti,ab,kw OR ‘information processing’:ti,ab,kw OR ‘learning’:ti,ab,kw OR ‘memory’:ti,ab,kw OR ‘mental function*’:ti,ab,kw OR ‘neural function’:ti,ab,kw OR ‘neurocognit*’:ti,ab,kw OR ‘neuropsycho*’:ti,ab,kw OR ‘oddball’:ti,ab,kw OR ‘order threshold’:ti,ab,kw OR ‘performance accuracy’:ti,ab,kw OR ‘performance speed’:ti,ab,kw OR ‘psychomotor’:ti,ab,kw OR ‘reaction time’:ti,ab,kw OR ‘response time’:ti,ab,kw OR ‘selective attention’:ti,ab,kw OR ‘sentence verification’:ti,ab,kw OR ‘simple reaction’:ti,ab,kw OR ‘spatial compatibility’:ti,ab,kw OR ‘spatial recognition’:ti,ab,kw OR ‘speed of processing’:ti,ab,kw OR ‘stroop’:ti,ab,kw OR ‘sustained attention’:ti,ab,kw OR ‘test battery’:ti,ab,kw OR ‘trail making’:ti,ab,kw OR ‘verbal fluency’:ti,ab,kw OR ‘verbal item’:ti,ab,kw OR ‘verbal performance’:ti,ab,kw OR ‘verification task’:ti,ab,kw OR ‘vigilance’:ti,ab,kw OR ‘visual discrimination’:ti,ab,kw OR ‘visual task’:ti,ab,kw OR ‘word recall’:ti,ab,kw)

AND (‘child*’:ti,ab,kw OR ‘adolescen*’:ti,ab,kw OR ‘adult*’:ti,ab,kw OR ‘elderly’:ti,ab,kw OR ‘human*’:ti,ab,kw OR ‘individual*’:ti,ab,kw OR ‘patient*’:ti,ab,kw OR ‘participant*’:ti,ab,kw OR ‘student*’:ti,ab,kw OR ‘subject*’:ti,ab,kw OR ‘volunteer*’:ti,ab,kw)

AND [embase]/lim AND ([article]/lim OR [article in press]/lim OR [data papers]/lim OR [review]/lim) AND [humans]/lim
