# Supplementary material for: The effect of exposure to radiofrequency electromagnetic fields on cognitive performance in human experimental studies: A protocol for a systematic review
Source: Environ Int. 2021 Dec;157:106783. doi: 10.1016/j.envint.2021.106783 (PMC8485020; doi:10.1016/j.envint.2021.106783)
Supplement: Supplementary data 3 [file mmc3.docx]

Scopus

( TITLE-ABS-KEY ( "Electromagnetic Radiation*" ) OR TITLE-ABS-KEY ( "Electromagnetic Wave*" ) OR TITLE-ABS-KEY ( "Electromagnetic Energ*" ) OR TITLE-ABS-KEY ( "Radio Wave*" ) OR TITLE-ABS-KEY ( radiowave* ) OR TITLE-ABS-KEY ( "Hertzian Wave*" ) OR TITLE-ABS-KEY ( "High Frequency Wave*" ) OR TITLE-ABS-KEY ( "Short Wave*" ) OR TITLE-ABS-KEY ( "Microwave Field*" ) OR TITLE-ABS-KEY ( "Microwave Radiat*" ) OR TITLE-ABS-KEY ( "Microwave Expos*" ) OR TITLE-ABS-KEY ( "Microwave Irradiat*" ) OR TITLE-ABS-KEY ( "Microwave Range*" ) OR TITLE-ABS-KEY ( "Micro Wave Field*" ) OR TITLE-ABS-KEY ( "Micro Wave Radiat*" ) OR TITLE-ABS-KEY ( "Micro Wave Expos*" ) OR TITLE-ABS-KEY ( "Micro Wave Irradiat*" ) OR TITLE-ABS-KEY ( "Micro Wave Range*" ) OR TITLE-ABS-KEY ( "MW Field*" ) OR TITLE-ABS-KEY ( "MW Radiat*" ) OR TITLE-ABS-KEY ( "MW Expos*" ) OR TITLE-ABS-KEY ( "MW Irradiat*" ) OR TITLE-ABS-KEY ( "MW Range*" ) OR TITLE-ABS-KEY ( "M W Field*" ) OR TITLE-ABS-KEY ( "M W Radiat*" ) OR TITLE-ABS-KEY ( "M W Expos*" ) OR TITLE-ABS-KEY ( "M W Irradiat*" ) OR TITLE-ABS-KEY ( "M W Range*" ) OR TITLE-ABS-KEY ( "EHF Wave*" ) OR TITLE-ABS-KEY ( "Ultrahigh Frequency Wave*" ) OR TITLE-ABS-KEY ( uhf ) OR TITLE-ABS-KEY ( radiofrequenc* ) OR TITLE-ABS-KEY ( "Radio Frequenc*" ) OR TITLE-ABS-KEY ( "RF Wave*" ) OR TITLE-ABS-KEY ( "RF Field*" ) OR TITLE-ABS-KEY ( "RF Electric Field*" ) OR TITLE-ABS-KEY ( "RF Magnetic Field*" ) OR TITLE-ABS-KEY ( "RF Radiation*" ) OR TITLE-ABS-KEY ( "RF Expos*" ) OR TITLE-ABS-KEY ( "RF EMF" ) OR TITLE-ABS-KEY ( "Millimeter Wave*" ) OR INDEXTERMS ( "Electromagnetic Fields" ) OR TITLE-ABS-KEY ( "Electromagnetic Environment*" ) OR TITLE-ABS-KEY ( "Electromagnetic Field*" ) OR TITLE-ABS-KEY ( "Electromagnetic Phenomen*" ) OR TITLE-ABS-KEY ( electromagnetics ) OR TITLE-ABS-KEY ( electromagnetism ) OR INDEXTERMS ( radar ) OR TITLE-ABS-KEY ( radar ) OR INDEXTERMS ( "Cell Phone" ) OR TITLE-ABS-KEY ( "Cell Phone*" ) OR TITLE-ABS-KEY ( cellphone* ) OR TITLE-ABS-KEY ( "Cellular Phone*" ) OR TITLE-ABS-KEY ( "Cellular Telephone*" ) OR TITLE-ABS-KEY ( "Mobile Phone*" ) OR TITLE-ABS-KEY ( "Mobile Telephone*" ) OR TITLE-ABS-KEY ( "Cordless Phone*" ) OR TITLE-ABS-KEY ( "Car Phone*" ) OR INDEXTERMS ( smartphone* ) OR TITLE-ABS-KEY ( "Smart Phone*" ) OR TITLE-ABS-KEY ( iphone* ) OR TITLE-ABS-KEY ( "i-Phone*" ) OR TITLE-ABS-KEY ( android ) OR INDEXTERMS ( "Cell Phone Use" ) OR INDEXTERMS ( "Wireless Technology" ) OR TITLE-ABS-KEY ( "Wireless Technolog*" ) OR TITLE-ABS-KEY ( "Wireless Communication*" ) OR TITLE-ABS-KEY ( "Wi-Fi" ) OR TITLE-ABS-KEY ( wifi ) OR TITLE-ABS-KEY ( "Specific Absorption Rate*" ) OR TITLE-ABS-KEY ( "W/kg" ) OR TITLE-ABS-KEY ( "Global System for Mobile Communication*" ) OR TITLE-ABS-KEY ( gsm ) OR TITLE-ABS-KEY ( "Digital Cellular System*" ) OR TITLE-ABS-KEY ( "Universal Mobile Telecommunication System*" ) OR TITLE-ABS-KEY ( umts ) OR TITLE-ABS-KEY ( "Code Division Multiple Access" ) OR TITLE-ABS-KEY ( cdma ) OR TITLE-ABS-KEY ( wcdma ) OR TITLE-ABS-KEY ( wimax ) OR TITLE-ABS-KEY ( bluetooth ) OR TITLE-ABS-KEY ( "Total Access Communication System" ) OR TITLE-ABS-KEY ( "Terrestrial Trunked Radio" ) OR TITLE-ABS-KEY ( tetra ) OR TITLE-ABS-KEY ( "Digital Enhanced Cordless Telecommunication*" ) )

AND ( TITLE-ABS-KEY ( "auditory task" ) OR TITLE-ABS-KEY ( "choice reaction" ) OR TITLE-ABS-KEY ( "clock monitoring" ) OR TITLE-ABS-KEY ( "contingent negative variation" ) OR TITLE-ABS-KEY ( "cognit*" ) OR TITLE-ABS-KEY ( "contingent negative variation" ) OR TITLE-ABS-KEY ( "critical flicker frequency" ) OR TITLE-ABS-KEY ( "critical fusion frequency" ) OR TITLE-ABS-KEY ( "decision making" ) OR TITLE-ABS-KEY ( "digit span" ) OR TITLE-ABS-KEY ( "discrimination task" ) OR TITLE-ABS-KEY ( "divided attention" ) OR TITLE-ABS-KEY ( "executive function*" ) OR TITLE-ABS-KEY ( "information processing" ) OR TITLE-ABS-KEY ( "learning" ) OR TITLE-ABS-KEY ( "memory" ) OR TITLE-ABS-KEY ( "mental function*" ) OR TITLE-ABS-KEY ( "neural function" ) OR TITLE-ABS-KEY ( "neurocognit*" ) OR TITLE-ABS-KEY ( "neuropsycho*" ) OR TITLE-ABS-KEY ( "oddball" ) OR TITLE-ABS-KEY ( "order threshold" ) OR TITLE-ABS-KEY ( "performance accuracy" ) OR TITLE-ABS-KEY ( "performance speed" ) OR TITLE-ABS-KEY ( "psychomotor" ) OR TITLE-ABS-KEY ( "reaction time" ) OR TITLE-ABS-KEY ( "response time" ) OR TITLE-ABS-KEY ( "selective attention" ) OR TITLE-ABS-KEY ( "sentence verification" ) OR TITLE-ABS-KEY ( "simple reaction" ) OR TITLE-ABS-KEY ( "spatial compatibility" ) OR TITLE-ABS-KEY ( "spatial recognition" ) OR TITLE-ABS-KEY ( "speed of processing" ) OR TITLE-ABS-KEY ( "stroop" ) OR TITLE-ABS-KEY ( "sustained attention" ) OR TITLE-ABS-KEY ( "test battery" ) OR TITLE-ABS-KEY ( "trail making" ) OR TITLE-ABS-KEY ( "verbal fluency" ) OR TITLE-ABS-KEY ( "verbal item" ) OR TITLE-ABS-KEY ( "verbal performance" ) OR TITLE-ABS-KEY ( "verification task" ) OR TITLE-ABS-KEY ( "vigilance" ) OR TITLE-ABS-KEY ( "visual discrimination" ) OR TITLE-ABS-KEY ( "visual task" ) OR TITLE-ABS-KEY ( "word recall" ) )

AND ( TITLE-ABS-KEY ( "child*" ) OR TITLE-ABS-KEY ( "adolescen*" ) OR TITLE-ABS-KEY ( "adult*" ) OR TITLE-ABS-KEY ( "elderly" ) OR TITLE-ABS-KEY ( "human*" ) OR TITLE-ABS-KEY ( "individual*" ) OR TITLE-ABS-KEY ( "patient*" ) OR TITLE-ABS-KEY ( "participant*" ) OR TITLE-ABS-KEY ( "student*" ) OR TITLE-ABS-KEY ( "subject*" ) OR TITLE-ABS-KEY ( "volunteer*" ))

AND ( LIMIT-TO ( DOCTYPE , "ar" ) OR LIMIT-TO ( DOCTYPE , "re" ) OR LIMIT-TO ( DOCTYPE , "dp" ) ) AND ( LIMIT-TO ( EXACTKEYWORD , "Human" ) OR LIMIT-TO ( EXACTKEYWORD , "Humans" ) )
