# Supplementary material for: The effect of exposure to radiofrequency electromagnetic fields on cognitive performance in human experimental studies: A protocol for a systematic review
Source: Environ Int. 2021 Dec;157:106783. doi: 10.1016/j.envint.2021.106783 (PMC8485020; doi:10.1016/j.envint.2021.106783)
Supplement: Supplementary data 4 [file mmc4.docx]

**Web of Science**

TS=("Electromagnetic Wave*" OR "Electromagnetic Energ*" OR "Electromagnetic Radiation*" OR "Radio Wave*" OR Radiowave* OR "Hertzian Wave*" OR "High Frequency Wave*" OR "Short Wave*" OR "Microwave Field*" OR "Microwave Radiat*" OR "Microwave Expos*" OR "Microwave Irradiat*" OR "Microwave Range*" OR "Micro Wave Field*" OR "Micro Wave Radiat*" OR "Micro Wave Expos*" OR "Micro Wave Irradiat*" OR "Micro Wave Range*" OR "MW Field*" OR "MW Radiat*" OR "MW Expos*" OR "MW Irradiat*" OR "MW Range*" OR "M W Field*" OR "M W Radiat*" OR "M W Expos*" OR "M W Irradiat*" OR "M W Range*" OR "EHF Wave*" OR "Ultrahigh Frequency Wave*" OR UHF OR Radiofrequenc* OR "Radio Frequenc*" OR "RF Wave*" OR "RF Field*" OR "RF Electric Field*" OR "RF Magnetic Field*" OR "RF Radiation*" OR "RF Expos*" OR "RF EMF" OR "Millimeter Wave*" OR "Electromagnetic Environment*" OR "Electromagnetic Field*" OR "Electromagnetic Phenomen*" OR Electromagnetics OR Electromagnetism OR Radar OR "Cell Phone*" OR Cellphone* OR "Cellular Phone*" OR "Cellular Telephone*" OR "Mobile Phone*" OR "Mobile Telephone*" OR "Cordless Phone*" OR "Car Phone*" OR Smartphone* OR "Smart Phone*" OR iPhone* OR i-Phone* OR Android OR "Wireless Technolog*" OR "Wireless Communication*" OR Wi-Fi OR Wifi OR "Specific Absorption Rate*" OR "W/kg" OR "Global System for Mobile Communication*" OR GSM OR "Digital Cellular System*" OR "Universal Mobile Telecommunication System*" OR UMTS OR "Code Division Multiple Access" OR CDMA OR WCDMA OR WiMAX OR Bluetooth OR "Total Access Communication System" OR "Terrestrial Trunked Radio" OR TETRA OR "Digital Enhanced Cordless Telecommunication*")

AND TS= ("auditory task" OR "choice reaction" OR "clock monitoring" OR "contingent negative variation" OR "cognit*" OR "contingent negative variation" OR "critical flicker frequency" OR "critical fusion frequency" OR "decision making" OR "digit span" OR "discrimination task" OR "divided attention" OR "executive function*" OR "information processing" OR "learning" OR "memory" OR "mental function*" OR "neural function" OR "neurocognit*" OR "neuropsycho*" OR "oddball" OR "order threshold" OR "performance accuracy" OR "performance speed" OR "psychomotor" OR "reaction time" OR "response time" OR "selective attention" OR "sentence verification" OR "simple reaction" OR "spatial compatibility" OR "spatial recognition" OR "speed of processing" OR "stroop" OR "sustained attention" OR "test battery" OR "trail making" OR "verbal fluency" OR "verbal item" OR "verbal performance" OR "verification task" OR "vigilance" OR "visual discrimination" OR "visual task"OR "word recall")

AND TS=("child*" OR "adolescen*" OR "adult*" OR "elderly" OR "human*" OR "individual*" OR "patient*" *" OR "participant*" OR "student*" OR "subject*" OR "volunteer)

and **Articles** or **Review** **Articles** or **Early** **Access** (Document Types)
