# Supplementary material for: The effect of exposure to radiofrequency electromagnetic fields on cognitive performance in human experimental studies: A protocol for a systematic review
Source: Environ Int. 2021 Dec;157:106783. doi: 10.1016/j.envint.2021.106783 (PMC8485020; doi:10.1016/j.envint.2021.106783)
Supplement: Supplementary data 5 [file mmc5.docx]

EMF-Portal

1. **Keywords** – "Or"
2. **Topics** – "Experimental studies," "Reviews, surveys, summaries,"
3. **Frequency ranges** – "Radio frequency (≥ 10 MHz)" and "Mobile communications"
4. **Time span** – "Complete time span"

**Searches:**

cognit* "executive function*" "information processing" learning memory "mental function*" "neural function*" neurocognit* neuropsycho*

"choice reaction" "discrimination task" "divided attention" "decision making" "vigilance" "test battery" "verification task"

"auditory task" "oddball" "order threshold" "clock monitoring" "contingent negative variation" "psychomotor" "visual perception"

"critical flicker frequency" "critical fusion frequency" "stroop" "visual discrimination" "visual task" "spatial compatibility" "spatial recognition" "trail making"

"performance accuracy" "performance speed" "reaction time" "response time" "speed of processing" "selective attention" "sustained attention"

"digit span" "simple reaction" "sentence verification" "verbal fluency" "verbal item" "verbal performance" "word recall"
