# Supplementary material for: The effect of exposure to radiofrequency electromagnetic fields on cognitive performance in human experimental studies: A protocol for a systematic review
Source: Environ Int. 2021 Dec;157:106783. doi: 10.1016/j.envint.2021.106783 (PMC8485020; doi:10.1016/j.envint.2021.106783)
Supplement: Supplementary data 7 [file mmc7.docx]

Experimental Studies of exposure to Radiofrequency EMF and cognitive impairment: Risk of bias analysis

**Selection bias:**

1. **Was administered exposure level adequately randomized?**

“Group” means subjects/persons/volunteers allocated to a fixed exposure condition or exposure condition sequence

| Definitely Low Risk of Bias (++) |
| --- |
| There is direct evidence that subjects were allocated to any study group including controls using a method with a random component.  Acceptable methods of randomization include: referring to a random number table, using a computer random number generator, coin tossing, shuffling cards or envelopes, throwing dice, or drawing of lots (Higgins and Green 2011). Restricted randomization (e.g., blocked randomization) to ensure particular allocation ratios will be considered low risk of bias. Similarly, stratified randomization and minimization approaches that attempt to minimize imbalance between groups will be considered acceptable. |
| Probably Low Risk of Bias (+) |
| There is indirect evidence that subjects were allocated to study groups using a method with a random component (i.e., authors state that allocation was random, without description of the method used),  **OR** it is deemed that allocation without a clearly random component during the study would not appreciably bias results.  For example, approaches such as biased coin or urn randomization, replacement randomization, mixed randomization, and maximal randomization may require consultation with a statistician to determine risk-of-bias rating (Higgins and Green 2011). |
| Probably High Risk of Bias (-) |
| There is indirect evidence that subjects were allocated to study groups using a method with a non-random component,  **OR** there is insufficient information provided about how subjects were allocated to study groups (record “NR” as basis for answer).  **Note:** Non-random allocation methods may be systematic but have the potential to allow participants or researchers to anticipate the allocation to study groups. Such “quasi-random” methods include alternation, assignment based on date of birth, case record number, or date of presentation to study (Higgins and Green 2011). |
| Definitely High Risk of Bias (--) |
| There is direct evidence that subjects were allocated to study groups using a non-random method including judgment of the researcher, preference of the participant, the results of a pre-test, or availability of the exposure set-up.  Non-randomized studies will be excluded before data extraction. This response will be not applicable for included studies. |

***2. Was allocation to study groups adequately concealed?***

| Definitely Low Risk of Bias (++) |
| --- |
| There is direct evidence that at the time of recruitment the research personnel and subjects did not know what study group subjects were allocated to, and it is unlikely that they could have broken the blinding of allocation until after recruitment was complete and irrevocable.  Acceptable methods used to ensure allocation concealment include central allocation (including telephone, web-based and externally controlled randomization) or equivalent methods. |
| Probably Low Risk of Bias (+) |
| There is indirect evidence that the research personnel and subjects did not know what study group subjects were allocated to and it is unlikely that they could have broken the blinding of allocation until after recruitment was complete and irrevocable,  **OR** it is deemed that lack of adequate allocation concealment would not appreciably bias results. |
| Probably High Risk of Bias (-) |
| There is indirect evidence that at the time of recruitment it was possible for the research personnel and subjects to know what study group subjects were allocated to, or it is likely that they could have broken the blinding of allocation before recruitment was complete and irrevocable,  **OR** there is insufficient information provided about allocation to study groups (record “NR” as basis for answer).  **Note:** Inadequate methods include using an open random allocation schedule (e.g., a list of random numbers); alternation or rotation; date of birth; case record number; or any other explicitly unconcealed procedure. |
| Definitely High Risk of Bias (--) |
| There is direct evidence that at the time of recruitment, it was possible for the research personnel and subjects to know what study group subjects were allocated to, or it is likely that they could have broken the blinding of allocation before recruitment was complete and irrevocable. |

**3. Is an equal proportion of participants allocated to any study group to prevent a period effect? (Only applicable for cross over trials)**

“Period effects” are defined as systematic changes in outcome over time that cannot be attributed to the different exposure conditions. These include for example order or learning effects.

| Definitely Low Risk of Bias (++) |
| --- |
| There is direct evidence that an equal proportion of subjects are allocated to any study group (counterbalancing)  **OR** there is direct evidence that period effects are included in the analysis. |
| Probably Low Risk of Bias (+) |
| There is indirect evidence that an equal proportion of subjects are allocated to any study group  **OR** there is indirect evidence that period effects are included in the analysis. |
| Probably High Risk of Bias (-) |
| There is indirect evidence that an unequal proportion of subjects are allocated to any study group  **AND** there is indirect evidence that period effects are NOT included in the analysis  **OR** there is insufficient information provided about allocation to study groups (NR). |
| Definitely High Risk of Bias (--) |
| There is direct evidence that an unequal proportion of subjects are allocated to any study group  **AND** there is direct evidence that period effects are NOT included in the analysis. |

**Performance bias**

**4. Were experimental conditions identical across study groups?**

| Definitely Low Risk of Bias (++) |
| --- |
| There is direct evidence that the same exposure-related conditions were used for EMF and sham application,  **AND** there is direct evidence that non-exposure-related experimental conditions (e.g. fixed time schedule, prior consumption of caffeine, nicotine, medication, prior EMF exposure) were identical across study groups (i.e., the study report explicitly provides this level of detail). |
| Probably Low Risk of Bias (+) |
| There is indirect evidence that the same exposure-related conditions were used for EMF and sham application,  **OR** it is deemed that the exposure-related conditions would not appreciably bias results.  **AND** as described above, identical non-exposure-related experimental conditions are assumed if authors did not report differences. |
| Probably High Risk of Bias (-) |
| There is indirect evidence that the exposure-related conditions differed between EMF and sham/control application,  **OR** authors did not report the exposure-related conditions used for EMF and sham/control applications (record “NR” as basis for answer),  **OR** there is indirect evidence that non-exposure-related experimental conditions were not comparable between study groups. |
| Definitely High Risk of Bias (--) |
| There is direct evidence from the study report that exposure-related conditions differed between EMF and sham/control applications,  **OR** there is direct evidence that non-exposure-related experimental conditions were not comparable between study groups. |

**5. Were the research personnel and human subjects blinded to the study group during the study?**

| Definitely Low Risk of Bias (++) |
| --- |
| There is direct evidence that the subjects and research personnel (data collector, data processor) were adequately blinded to study group, and it is unlikely that they could have broken the blinding during the study. Methods used to ensure blinding include automated application of exposure, encrypted recording of exposure-related data on the control computer, and taking all necessary measures to prevent indirect perception/detection of exposure by the subjects and/or the research personnel. |
| Probably Low Risk of Bias (+) |
| There is indirect evidence that the research personnel and subjects were adequately blinded to study group, and it is unlikely that they could have broken the blinding during the study,  **OR** it is deemed that lack of adequate blinding during the study would not appreciably bias results. |
| Probably High Risk of Bias (-) |
| There is indirect evidence that it was possible for research personnel or subjects to infer the study group,  **OR** there is insufficient information provided about blinding to study group during the study (record “NR” as basis for answer). Note: Inadequate methods include using an open random allocation schedule (e.g., a list of random numbers), alternation or rotation; date of birth; case record number; any other explicitly unconcealed procedure. Moreover, insufficient information about countermeasures against indirect perception/detection of exposure will also be considered as “Probably High Risk of Bias” |
| Definitely High Risk of Bias (--) |
| There is direct evidence for lack of adequate blinding of the study group including no blinding or incomplete blinding of research personnel and subjects. |

**6. Was there sufficient time for any carry-over effects in cross-over studies to have disappeared before outcome assessment in the second period? (Only applicable for cross over trials)**

| Definitely Low Risk of Bias (++) |
| --- |
| There is direct evidence that there was a sufficiently long wash-out or recovery period between the treatments to allow for a complete recovery from the previous exposure and to exclude any carry-over effects (24h and more (Regel and Achermann 2011)). This means, the subjects have reached the same baseline at the beginning of each experiment. |
| Probably Low Risk of Bias (+) |
| There is indirect evidence there was a sufficiently long wash-out or recovery period between the treatments to allow for a complete recovery from the previous exposure an exclude any carry-over effects (24h and more). |
| Probably High Risk of Bias (-) |
| There is indirect evidence that the wash-out period (recovery period) was absent or too short for carry-over effects to have disappeared (e.g. there was a washout period between the application of different exposure levels which appeared to be sufficient, but the baseline at the beginning of the experiments differed between groups/exposure levels)  **OR** there is no information about the washout period. |
| Definitely High Risk of Bias (--) |
| There is direct evidence that the wash-out period (recovery period) was absent or too short for carry-over effects to have disappeared (experiments at several exposure levels within 24h). |

**Attrition / Exclusion bias**

**7. Were outcome data complete without attrition or exclusion from analysis?**

| Definitely Low Risk of Bias (++) |
| --- |
| There is direct evidence that there was no loss of subjects during the study and outcome data were complete,  **OR** loss of subjects (i.e., incomplete outcome data) was adequately addressed and reasons were documented when human subjects were removed from a study or analyses. Review authors should be confident that the participants included in the analysis are exactly those who were randomized into the trial. Acceptable handling of subject attrition includes: very little missing outcome data (less than 20% in each group (Genaidy et al. 2007)); reasons for missing subjects unlikely to be related to outcome; missing outcome data balanced in numbers across study groups, with similar reasons for missing data across groups,  **OR** analyses (such as intention-to-treat analysis) in which missing data have been imputed using appropriate methods (insuring that the characteristics of subjects lost to follow up or with unavailable records are described in identical way and are not significantly different from those of the study participants).  **Note:** Participants randomized but subsequently found not to be eligible need not always be considered as having missing outcome data (Higgins and Green 2011). |
| Probably Low Risk of Bias (+) |
| There is indirect evidence that loss of subjects (i.e., incomplete outcome data) was adequately addressed and reasons were documented when human subjects were removed from a study,  **OR** it is deemed that the proportion lost to follow-up would not appreciably bias results (less than 20% in each group (Genaidy et al. 2007)). This would include reports of no statistical differences in characteristics of subjects lost to follow up or with unavailable records from those of the study participants. Generally, the higher the ratio of participants with missing data to participants with events, the greater potential there is for bias. For studies with a long duration of follow-up, some withdrawals for such reasons are inevitable. |
| Probably High Risk of Bias (-) |
| - There is indirect evidence that loss of subjects (i.e., incomplete outcome data) was unacceptably large (greater than 20% in each group (Genaidy et al. 2007)) and not adequately addressed, - **OR** there is insufficient information provided about numbers of subjects lost to follow-up (record “NR” as basis for answer). |
| Definitely High Risk of Bias (--) |
| There is direct evidence that loss of subjects (i.e., incomplete outcome data) was unacceptably large and not adequately addressed. Unacceptable handling of subject attrition includes: reason for missing outcome data likely to be related to true outcome, with either imbalance in numbers or reasons for missing data across study groups; or potentially inappropriate application of imputation. |

**Detection bias**

**8. Can we be confident in the exposure characterization and is the exposure contrast sufficient?**

| Definitely Low Risk of Bias (++) |
| --- |
| There is direct evidence that exposure was consistently generated with the same well controlled exposure apparatus for all considered exposure levels, and that the resulting exposure of the subjects were assessed using well-established methods that are directly related to SAR level inside the brain,  **OR** exposure was assessed using less-established methods that are validated against SAR models that represent the investigated exposure situation.  **AND** the exposure distribution inside the brain contains sufficient contrast between the lowest and the highest exposure categories, when taking into account intra- and inter-individual variations of exposure (in terms of SAR).  “Well-established” exposure assessment methods in the above sense include appropriate numerical computations of SAR inside the brain of anatomical body models using validated numerical source models. For localized exposure of the head with the radiation source close to the ear, only indicating maximum spatial average SAR (SAR10g, SAR1g) in the entire head (i.e., including ear tissue) is considered insufficient, as the SAR1g and/or the SAR10g value may be highly dominated by the absorption inside the ear and can therefore not be seen as a reliable proxy for brain exposure. Similarly, only indicating SAR10g or SAR1g assessed in a homogeneous head phantom is considered insufficient in case of localized exposure of the head with the radiation source close to the ear.  “Less-established” exposure assessment methods in the above sense include the assessment of exposure by the undisturbed (i.e. measured in absence of the subject) external electric or magnetic field strengths (or power flux density) at the site of the subject, which can be transferred to SAR levels in case of far field conditions. Only reporting external field quantities in case of near field exposure is considered insufficient.  “Sufficient exposure contrast” means that the level of exposure in “exposed” condition is higher than the exposure level of the control condition plus expanded uncertainty (CI 95%) of the exposure level assessment, suggesting that actual exposure levels of different treatments do not overlap. |
| Probably Low Risk of Bias (+) |
| There is indirect evidence that the exposure was consistently generated and assessed in the sense described above  **AND** the exposure distribution inside the brain contains sufficient contrast between the lowest and the highest exposure categories, when taking into account intra- and inter-individual variations of exposure (in terms of SAR). |
| Probably High Risk of Bias (-) |
| There is indirect evidence that the exposure was generated and assessed using poorly validated methods (e.g. indicating SAR1g or SAR10g values inside homogeneous head phantom in case of localized exposure of the head with the radiation source close to the ear),  **OR** there is insufficient information provided about the exposure generation and assessment, but no evidence for concern about the method used (record “NR” as basis for answer).  **OR** the exposure contrast is probably too low to ensure a non-overlapping of lowest and highest exposure categories when taking into account intra- and inter-individual variations of exposure (in terms of SAR). |
| Definitely High Risk of Bias (--) |
| There is direct evidence that exposure generation and assessment were done with poorly validated methods (e.g. indicating SAR1g or SAR10g values inside homogeneous head phantom in case of localized exposure of the head with the radiation source close to the ear; lack of control/monitoring of exposure),  **OR** there is direct evidence that the exposure contrast is too low to ensure a non-overlapping of lowest and highest exposure categories when taking into account intra- and inter-individual variations of exposure (in terms of SAR).  Studies reporting only tissue-external exposure metrics will also be rated definitely high risk of bias*.* |

**9. Can we be confident in the outcome assessment?**

| Definitely Low Risk of Bias (++) |
| --- |
| There is direct evidence that the outcome was assessed using well-established methods (e.g., the “gold standard” with validity and reliability >0.70 (Genaidy et al. 2007)) that capture a specific/known cognitive domain (e.g. reference to a publication of the specific test/task is available and/or reference to a functionally equivalent is provided).  **AND** assessment methods for cognitive performance outcome are standardized, whereas standardization refers  to the consistency of instruction, conduction, scoring and interpretation of results across all study participants (task specific: number of (sub-)tests, sensory modality tested, level of task difficulty, task duration, task order, mode of presentation – paper-pencil vs. digital, comparability of parallel test versions, practice session).  **AND** performance outcome was measured in terms of accuracy (correct, false or and missed responses) and/or speed (reaction time) or other standardized qualitative outcome parameter (e.g. angular deviation)  **AND** there is direct evidence that the outcome assessors (including study subjects, if outcomes were self-reported) were adequately blinded to the study group, and it is unlikely that they could have broken the blinding prior to reporting outcomes. |
| Probably Low Risk of Bias (+) |
| There is indirect evidence that the outcome was assessed using acceptable methods (i.e., deemed valid and reliable but not the gold standard, with validity and reliability ≥0.40, Genaidy et al. 2007), that capture a specific/known cognitive domain, but no reference to a publication of the specific test/task/test battery is available and/or reference to a functionally equivalent is provided.  **AND** assessment methods for cognitive performance outcome are standardized (see above).  **AND** there is indirect evidence that the outcome assessors (including study subjects) were adequately blinded to the study group, and it is unlikely that they could have broken the blinding prior to reporting outcomes,  **OR** it is deemed that lack of adequate blinding of outcome assessors would not appreciably bias results, which is more likely to apply to objective outcome measures. |
| Probably High Risk of Bias (-) |
| There is indirect evidence that the outcome assessment method is an insensitive instrument (e.g., a cognitive test/task used to assess outcomes with no information on validation or on other test criteria and there are no references to former publications on the test/task available).  **AND** assessment methods for cognitive performance outcome were standardized (see above).  **OR** there is indirect evidence that the outcome assessors (including study subjects) were adequately blinded to the study group, and it is unlikely that they could have broken the blinding prior to reporting outcomes,  **OR** there is insufficient information provided about blinding of outcome assessors (record “NR” as basis for answer). |
| Definitely High Risk of Bias (--) |
| There is direct evidence that the outcome assessment method is an insensitive instrument, and no information on test/task procedure and outcome parameter are reported.  **OR** there is direct evidence for lack of adequate blinding of outcome assessors (including study subjects if outcomes were self-reported), including no blinding or incomplete blinding. |

***Selective reporting bias***

***10. Were all measured outcomes reported?***

| Definitely Low Risk of Bias (++) |
| --- |
| There is direct evidence that all of the study’s measured outcomes (primary and secondary) outlined in the protocol, methods, abstract, and/or introduction, or mentioned in the discussion (that are relevant for the evaluation) have been reported. This would include outcomes reported with sufficient detail to be included in meta-analysis or fully tabulated during data extraction and analyses had been planned in advance. |
| Probably Low Risk of Bias (+) |
| There is indirect evidence that all of the study’s measured outcomes (primary and secondary) outlined in the protocol, methods, abstract, and/or introduction, or mentioned in the discussion (that are relevant for the evaluation) have been reported,  **OR** analyses that had not been planned in advance (i.e., retrospective unplanned subgroup analyses) are clearly indicated as such and deemed that unplanned analyses were appropriate and selective reporting would not appreciably bias results (e.g., appropriate analyses of an unexpected effect). This would include outcomes reported with insufficient detail such as only reporting that results were statistically significant (or not). |
| Probably High Risk of Bias (-) |
| There is indirect evidence that all of the study’s measured outcomes (primary and secondary) outlined in the protocol, methods, abstract, and/or introduction, or mentioned in the discussion (that are relevant for the evaluation) have not been reported,  **OR** and there is indirect evidence that unplanned analyses were included that may appreciably bias results,  **OR** there is insufficient information provided about selective outcome reporting (record “NR” as basis for answer). |
| Definitely High Risk of Bias (--) |
| There is direct evidence that all of the study’s measured outcomes (primary and secondary) outlined in the protocol, methods, abstract, and/or introduction (that are relevant for the evaluation) have not been reported. In addition to not reporting outcomes, this would include outcomes reported using measurements, analysis methods or subsets of the data that were not pre-specified or reporting outcomes not pre-specified, or that unplanned analyses were included that would appreciably bias results. |

We will assign an item as definitely low risk of bias, if it is explicitly described how a possible bias was avoided, (e.g. measures to ensure a proper randomization, concealment, blinding etc. are explicitly described). We will assign an item as probably low risk of bias, if it is stated, for example, that the studies were randomized, cross-over, double-blinded etc., but it is not described how it was achieved. We will judge an item as probably high risk of bias if, based on the study description, there is a doubt that the criteria were fulfilled, or no information is given (in that case, we will contact the authors). An item will be rated as definitely high risk of bias if it is evident from the study description that a certain criterion has not been taken into account (e.g. the study was not double-blinded, the allocation not concealed, exposure level not defined, outcomes not completely reported).

We will try to judge the direction of bias when possible.
